# Obesity: Epidemiology, Pathophysiology, and Therapeutics

**DOI:** 10.3389/fendo.2021.706978

**Published:** 2021-09-06

**Authors:** Xihua Lin, Hong Li

**Affiliations:** Department of Endocrinology, Sir Run Run Shaw Hospital, School of Medicine, Zhejiang University, Hangzhou, China

**Keywords:** obesity, epidemiology, pathophysiology, genetics, epigenetics, microenvironment

## Abstract

Obesity is a complex multifactorial disease that accumulated excess body fat leads to negative effects on health. Obesity continues to accelerate resulting in an unprecedented epidemic that shows no significant signs of slowing down any time soon. Raised body mass index (BMI) is a risk factor for noncommunicable diseases such as diabetes, cardiovascular diseases, and musculoskeletal disorders, resulting in dramatic decrease of life quality and expectancy. The main cause of obesity is long-term energy imbalance between consumed calories and expended calories. Here, we explore the biological mechanisms of obesity with the aim of providing actionable treatment strategies to achieve a healthy body weight from nature to nurture. This review summarizes the global trends in obesity with a special focus on the pathogenesis of obesity from genetic factors to epigenetic factors, from social environmental factors to microenvironment factors. Against this background, we discuss several possible intervention strategies to minimize BMI.

## Background

There has been a significant global increase in obesity rate during the last 50 years. Obesity is defined as when a person has a body mass index [BMI (kg/m^2^), dividing a person’s weight by the square of their height] greater than or equal to 30, overweight is defined as a BMI of 25.0-29.9. Being overweight or obesity is linked with more deaths than being underweight and is a more common global occurrence than being underweight. This is a global phenomenon occurring in every region except parts of sub-Saharan Asia and Africa (1), and also countries with low obesity rates (i.e., Sri Lanka, Indonesia, Sudan, Singapore, Djibouti, etc.) (2).

Obesity increases the likelihood of various diseases and conditions which are linked to increased mortality. These include Type 2 diabetes mellitus (T2DM), cardiovascular diseases (CVD), metabolic syndrome (MetS), chronic kidney disease (CKD), hyperlipidemia, hypertension, nonalcoholic fatty liver disease (NAFLD), certain types of cancer, obstructive sleep apnea, osteoarthritis, and depression (3).Treating these conditions can place an additional load on healthcare systems: for example, it is estimated that obese have a 30% higher medical cost than those with a normal BMI (4). As related total health-care costs double every decade, treating the consequences of obesity poses an expensive challenge for patients (5).

There are several possible mechanisms leading to obesity. Actually, the traditional view is usually that the main cause is the significantly more excess energy stored than the energy the body used. The excess energy is stored in fat cells, thereby developing the characteristic obesity pathology. The pathologic enlargement of fat cells will alter the nutrient signals responsible for obesity (6).However, the latest research showed that the food sources and quality of nutrients matter more than their quantities in the diet for weight control, and also for disease prevention (7). More and more etiologies or defects that lead to obesity can be identified under the background of struggle between nurture and nature, genetic and epigenetic, environmental and microenvironment. We are increasingly understanding how food cravings are upregulated in obesity individuals’ brains, how gut hormones, adipose tissue, or gut microbiota regulate appetite and satiety in the hypothalamus, as well as the roles of gut dysbiosis played in obesity development and how dysfunction of glucose and lipids metabolism causes secondary health problems (8). In addition, genetic factors are known to play critical roles in determining an individual’s predisposition to weight gain (9). Recent epigenetic studies have provided very useful tools for understanding the worldwide increase in obesity (10). Studies have discussed the relationships between genetics, epigenetics, and environment in obesity and explored the roles of epigenetic factors in metabolism regulation and obesity risk as well as its complications (11).

The field of obesity is rapidly evolving as an abundance of new scientific data continue to emerge. Herein, we discuss the epidemiology of obesity, covering the pathophysiology, pathogenesis, genetics, epigenetics, and environmental (macro and micro) causes that result in obesity. We end by summarizing possible management and prevention strategies.

## Epidemiology of Obesity

BMI is used to define and diagnose obesity according to World Health Organization (WHO) guidelines (4). In adults, WHO defines ‘overweight’ as a BMI of 25.0 to 29.9 and ‘obese’ as a BMI ≥ 30.0. Obesity is further classified into three severity levels: class I (BMI 30.0-34.9), class II (BMI 35.0-39.9) and class III (BMI ≥ 40.0) (12).However, large individual differences exist in the percent body fat for the given BMI value, which can be attributed to sex, ethnicity and age (13).Excess fat deposition in the abdominal region is termed ‘abdominal obesity’ and is associated with greater health risks (14).The definition and measurement guidelines of abdominal obesity differed from WHO, IDF (International Diabetes Federation) to AHA (American Heart Association) (15). However, there is no international standard suitable for all countries or regions.

The prevalence of excessive weight gain has doubled worldwide since 1980, and about a third of the global population has been determined to be obese or overweight (16). Obesity rate has dramatically enhanced in both male and female, and across all ages, with proportionally higher prevalence in older persons and women (4). While this trend is present globally, absolute prevalence rates vary across regions, countries, and ethnicities. The prevalence of obesity also varies with socioeconomic status, with slower rates of BMI increase in high-​income and some middle-income countries. While obesity was once considered a problem of high-income countries, the incidence rates of obese or overweight children in high-​income countries, including the United States, Sweden, Denmark, Norway, France, Australia and Japan, have decreased or plateaued since the early 2000s (17).

In low- and middle-income countries, rates of overweight and obesity are rising especially in urban areas. In China, one study based on 12,543 participants monitored over 22 years revealed that the prevalence of age-adjusted obesity rose from 2.15% to 13.99% in both sexes, going from 2.78 to 13.22% in female and from 1.46 to 14.99% in male, respectively (18, 19). The overweight rate of African children under 5 years old has increased by 24% since 2000. As of 2019, almost half of the Asian children under 5 years old were obese or overweight (20). WHO datasets from sub-Sarahan Africa reveal that prevalence of overweight and obese in adults and stunting, underweight, and wasting in children are inversely associated (21).

## Pathogenesis of Obesity

The pathogenesis of obesity involves regulation of calorie utilization, appetite, and physical activity, but have complex interactions with availability of health-care systems, the role of socio-economic status, and underlying hereditary and environmental factors.

### Food Intake and Energy Balance

The essential causes of obesity remain somewhat controversial. Current health recommendations to manage obesity are based on the underlying physiological property that fat accumulation is driven by an energy imbalance between consumed and expended calories. The obesity epidemic has been fueled in large part by increased energy from greater availability of highly rewarding and energy-dense food. Diet and various social, economic, and environmental factors related to food supply have a significant effect on patient’s ability to achieve the balance (22). In a 13-year follow-up study on 3,000 young, those who consumed much more fast-food were found to weigh an average of ~6kg more and have larger waist circumferences than those with the lowest fast-food-intake. They were also found to have higher incidences of negative weight-related health issues, such as elevated triglycerides and twice the odds of developing MetS (23). These issues are compounded in certain individuals that possess a genetic susceptibility to fat accumulation, which may be caused by significant interactions between homeostatic circuits and brain reward. Accumulation of lipid metabolites, inflammatory signaling, or other hypothalamic neuron impairing mechanisms may also lead to obesity, which might explain the biological defense of elevated body fat mass (24).

Obesogenic marketing to promote beverages or foods that are high in sugar and fat negatively modulates human behavior. Such advertisements may increase preference for energy-​dense foods and beverages (25). Analysis showed that African American programs had more food advertisements than other general market programs. More food advertisements were for meat, candy, soda, and fast food than for grains, pasta, cereals, vegetables, and fruits. Advertised products were designed to be cheap, have a long shelf-life, and taste ‘irresistible’. This applies particularly to high-fat, high-sugar junk foods that can stimulate the brain reward centers, the same part of the brain that’s stimulated by cocaine, heroin, and other addictive drugs, that is, these products are specifically engineered to be addictive (25). The brain reward offers a plausible mechanism to explain the elevated body fat mass, however, it seems that only certain individuals present this characteristic according to this theory.

For clinicians, a systematic evaluation of patient health factors affecting energy intake, metabolism, and expenditure is required for effective management of obesity. However, attempting to manage obesity through behavioral alterations aiming at addressing these three factors is more often than not unsuccessful. This suggests that our understanding of energy management and the interactions between intake, metabolism, and expenditure are not yet fully understood (26).

### Family History and Lifestyle

Family history, lifestyle, and psychological factors all function in propensity for obesity. The likelihood of becoming obese can be affected by nature and nurture, enhanced by family genetics (propensity to accumulate fat) (27) or life style (poor dietary or exercise habits) (28). A child with one obese parent has a three-time risk to become obese as an adult, while when a child’s parents are both obese, this child has a 10-fold risk of future obesity. A cross-sectional observational study of 260 children (139 female, 121 male, aged 2.4 and 17.2 years) demonstrated that the family history of cardiometabolic diseases and obesity are critical risk factors for severity of obesity in childhood (29).

A prospective survey of 3148 school boys (aged six to ten years) in Ariana highlighted several child obesity risk factors, including parental obesity of parents, the snacks between meals especially after the dinner, lack of sleep (< 8 hours), and daily consumption of juice, sparkling drink, sweets, and sugary foods (30). Two studies of mother-child pairs in the United States found that the healthy lifestyle of mothers during the childhood and adolescence of their offspring was closely associated with a significantly reduced risk of obesity in their children (31). These results underscore the benefits of intervening at the family- or parental-level to reduce the risk of obesity in children (31).

However, parents are not the sole instigators of childhood obesity. For example, in the United States, physical education was used as a regular part of a public education curriculum (32). Starting 2011 physical education programs were curtailed such that 25 percent of students could achieve four out of five the national standards of at least 225 minutes weekly at the senior school levels and at least 150 minutes weekly at the primary level (33). Other factors that may have resulted in the decline of physical activity in children include increasing time spent on video game consoles and mobile devices at a reduction of time spent actively or outdoors. It is hard to argue against technological progress, but based on these studies, such innovations may be taking a toll on children’s health (34).

### Microenvironment and Gut Microbiome

Our knowledge of the intestinal microbiome has grown substantially over recent years, as has our understanding of its intricate relationship to disease. For example, obesity is involved in an altered gut microenvironment that supports more diverse viral species than found in leaner hosts (35). This environment is more susceptible to the generation of pathogenic variants that can induce more serious disease (36). Increasing evidence shows that variations of gut microbiome cause alterations in host weight and metabolism. For example, compared with those with normal gut microbiota, germ-free male mice (without gut microflora) had 42% less total body fat, even while consuming 29% more food a day. However, after cecal microbe colonization, the total body fat of these mice increased 57%in, lean body mass decreased 7%, and daily food intake decreased 27% (35). A follow-up study suggested that these alterations resulted from decreased metabolic rates, with concomitant increased adipose tissue deposition, as capillary density in distal small intestinal villi increased 25% after microflora colonization. Similar results were also observed from female mice (37).

The human body contains around 3.8 × 10^13^ microorganisms and the majority of them occupy the gastrointestinal tract. Over half of the microbial population are bacteria, followed by *Archaea* and *Eukarya* (38). The diversity of healthy gut microbiome allows for functional redundancy, in which multiple microbes can perform similar functions. Normally, gut microbiota have substantial beneficial roles in the host, including involvement in metabolism of carbohydrate and lipid, synthesis of vitamins and amino acids, epithelial cell proliferation, protection against pathogens, and hormone modulation. Gut bacteria can also break down indigestible molecules such as human milk oligosaccharides and plant polysaccharides (39). Imbalance of microbial populations (‘dysbiosis’) has been show to associate with a wide range of diseases including neurological disorders, inflammatory bowel disease, malnutrition, cancer, diabetes, and obesity (40). Recent research suggests that caloric restriction can beneficially reshape the gut microbiome and that antibiotic use can negatively harm gut microflora in ways that result in diabetes and obesity. Human studies support findings that microbiome alterations are associated with obesity; however, the exact mechanisms (i.e., ratios and amounts of microflora diversity) are still unknown (41).

Gut microbiota are central players in the host immune system. Disturbances in gut microflora can lead to inflammation of the intestinal lining (42). This response has been demonstrated to be mediated by TLRs (toll-like receptors), which identify and attack host microbes. For example, TLR4 recognizes the bacterial LPS (lipopolysaccharides) in the cell walls of Gram-negative bacteria while TLR5 recognizes bacterial flagellin. The body mass of TLR5-knockout mice increased 20% and their epididymal fat pad size increased 100% when compared to the wild-type controls (43). The dietary fiber and starch fermentation in lower gastrointestinal tract induced by microbiome can also produce SCFAs (Short-chain fatty acids), which can regulate production of gut hormone such as peptide YY (PYY) in the intestinal epithelium and GLP-1, GLP-2 (glucagon-like peptides), and the secretion of gastric inhibitory peptides by K cells (44). In obese patients, enzymes participated in or glucose signaling pathways are downregulated. It may be that alterations in specific microbial populations are more important than overall phylogenetic ratios, resulting in alterations in enzymes and SCFAs production, which further influence regulation of insulin and glucose, ultimately leading to development of obesity (41).

### Genetic Factors and Causes

The studies from family and twin studies showed that around 40-70% of the obesity variation in human are resulted from genetic factors (45). While during the last 20 years, environmental alterations have increased obesity rates, the genetic factors play key roles in development of obesity (46). GWAS (Genome-wide association scans) approaches have identified over 400 genes associated with T2DM (47, 48), however, these genes only predict 5% of obesity risk (49). The low predictive power may be due to the situation that gene-gene, gene-environment, and epigenetic interactions have not been thoroughly identified using the current methods based on population genetics (50). Many obesity -associated genes have been identified to be involved in energy homeostasis regulating pathways.

Genetic causes of obesity can be broadly classified as: 1) monogenic causes that result from a single gene mutation, primarily located in the leptin- melanocortin pathway. Many of the genes, such as AgRP (Agouti-related peptide), PYY (orexogenic), or MC4R (the melanocortin-4 receptor), were identified for monogenic obesity disrupt the regulatory system of appetite and weight, hormonal signals (ghrelin, leptin, insulin) are sensed by the receptors located in the arcuate nucleus of the hypothalamus (51). 2) Syndromic obesity were severe obesity results from neurodevelopmental abnormalities and other organ/system malformations. This may be caused by alterations in a single gene or a larger chromosomal region encompassing several genes (52). 3) Polygenic obesity is caused by cumulative contribution of many genes. Further, some people with obesity gain excess weight due to the multiple genes they have (53), and these genes make them to favor food and thereby have a higher caloric intake. The presence of these types of genes can cause increased caloric intake, increased hunger levels, reduced control overeating, reduced satiety, increased tendency to store body fat, and increased tendency to be sedentary (54).

Rare single-gene defects are associated with high level of hunger and can cause dramatic obesity in young children. Those individuals with severe obesity developed before two years old should consult obesity medicine specialists and consider to be involved in screening for MC4R Deficiency, leptin deficiency, and POMC deficiency (55). Leptin deficiency can cause diet-induced obesity and metabolic dysregulation. About 50% of female with polymorphism came up with binge eating (56). The MC4R polymorphism influences the release of ghrelin (57). The chromosome 2p22 (a region encompassing the POMC gene) has been identified as the site of gene(s) affecting obesity and obesity-related traits (58). These studies suggest that childhood obesity should be considered in the light of both environmental context and genetic heritage (59).

There are several genetic, neuroendocrine, and chromosomal precursors that can result in obesity. PWS (Prader-Willi Syndrome) is a neurodevelopmental disorder with hypothalamic dysfunction, due to the deficiency of imprinted genes (60). Endocrine disorders such as PCOS (Polycystic Ovary Syndrome) can also lead to increased body fat (61). Chromosomal defects can lead to obesity, including deletion of 16p11.2, 2q37 (brachydactyly mental retardation syndrome; BDMR), 1p36 (monosomy 1p36 syndrome), 9q34 (Kleefstra syndrome), 6q16 (PWS-like syndrome), 17p11.2 (Smith Magenis syndrome; SMS), and 11p13 (WAGR syndrome) (62). These conditions rely on the conventional current health recommendations that energy imbalance between calories consumed and expended is the key cause of obesity and present circumstances under which traditional weight management methods may not help.

### Epigenetic Modification

We have been able to identify some of the genes that contribute to monogenic forms of obesity, but the human genome alterations on timescales that are too long for the genome to be a major player in the current obesity pandemic. Epigenetics, however, may offer a logical explanation for increasing obesity prevalence over the past few decades without necessitating a radical change in the genome (63). In multicellular organisms, the genetic code is homogenous throughout the body, but the expression of code can vary across cell types. Epigenetics studies showed that the heritable regulatory alterations in the genetic expression do not require alterations in the nucleotide sequence (64). Epigenetic modifications can be thought of as the differential packaging of the DNA that either allows or silences the expression of certain genes across tissues. Environmental and gut microbiota can influence the epigenetic programming of parental gametes, or programming in later stages of life (10).

The known epigenetic mechanisms include DNA methylation, histone modifications, and miRNA-mediated regulation. These can be passed from one generation to another meiotically or mitotically. There is evidence showing that the perinatal and embryo-fetal development period plays a critical role in human tissues and organs programming (65). DNA methylation appears to be the most important epigenetic mechanism for regulating gene expression. Alterations in DNA methylation can be a hallmark of many diseases such as cancers (66). LEP (Leptin) plays critical roles in adipose tissue regulation. The maternal metabolic status can affect DNA methylation of LEP profile at birth, affecting metabolic remodeling of obesity (67). The Adiponectin (ADIPOQ) epigenetic status also has relationship with obesity, and association has been reported between LDL-cholesterol levels and DNA methylation of both LEP and ADIPOQ (68). Paternal obesity has also been associated with inhibited methylation levels in IGF2 (insulin-like growth factor 2) regions, which promote the division and growth of various types of cells (69). Other genes investigated in the context of metabolism and obesity include: tumor necrosis factor (TNF), hypoxia-inducible factor 3a (HIF3A), neuropeptide Y (NPY), insulin receptor substrate 1 (IRS1), mitochondrial transcription factor A (TFAM), interleukin 6 (IL6), lymphocyte antigen 86 (LY86) and glucose transport 4 (GLUT4) (10, 63).

Histones are proteins function in DNA packaging and modifications to histones are associated with epigenetic regulation of adipogenesis and obesity development (70). Five key regulatory genes in adipogenesis, CCAAT-enhancer-binding protein β (C/EBP β), pre-adipocyte factor-1 (Pref-1), adipocyte protein 2 (aP2), PPARγ, and C/EBPα, are modulated by histone modifications during adipocyte differentiation (71). The enzymes play roles in histone modification also function in obesity. They also regulate the expression of HDACs (histone deacetylases), which participated in the epigenetic control of gene expression involved in a large amount of environmental factors (72).

MicroRNAs (miRNAs) are 18 to 25 nucleotides long short noncoding RNA sequences that can regulate gene expression by gene silencing and post-transcriptional alterations. MicroRNAs function in a variety of biological processes, including adipocyte differentiation and proliferation, and are associated with low-grade inflammation and insulin resistance displayed in obese individuals (73). Increased levels of miRNAs including miR-486-3p, miR-142-3p, miR-486-5p, miR-423-5p and miR-130b were seen in children with high BMI values, among which 10 miRNAs exhibited significant alterations with increasing body weight (74). Zhao et al. identified miRNA as a signature for weight gain and showed that the individuals with a high-risk score for 8 of these miRNAs had over 3-fold higher odds of weight gain (75). Alterations in adipocyte-derived exosomal miRNAs is also seen following weight loss and decrease in insulin resistance after gastric bypass (76). miRNAs have been shown to play a key role in obesity and that the associated metabolic alterations can serve as biomarkers, or potentially therapeutic targets for intervention. Consideration of genetic and epigenetic causes of obesity provide valuable tools for the clinical treatment of obesity.

## Therapeutics of Obesity

### Lifestyle Modifications

Given the lack of specific pharmacological interventions, ‘lifestyle modification’ remains the cornerstone of obesity management (4). Individuals with obesity are suggested to lose at least 10% body weight *via* combination of diet, physical activity, and behavior therapy (or lifestyle modification) (77). Significant short-term weight loss can be achieved by consumption of portion-controlled diets (78). Long-term weight control can be achieved *via* high levels of physical activity and continued patient–practitioner contact. In many cases, lifestyle modification results in dramatic loss of body weight, leading to significant reduction of cardiovascular risk (79).

Since food choices are mainly determined by peoples’ surroundings, it is imperative that governments improve policies and environment to reduce the availability of unhealthful foods and make healthy foods more accessible. Policies should be changed to increase development of foods with reduced sugar, fat, and salt and decrease availability of obesogenic foods aimed at children (80). Policy makers and Practitioners must be made aware of the potential impact of food advertisements on human health and behavior and should encourage food manufacturers to create and promote weight-friendly foods. Nutrition educators should help teach how to evaluate food advertisements (81). Interventions aimed at motivating behavioral alterations (e.g., health promotion, nutrition education, incentives for healthy living, sugar-​sweetened beverage tax, and social marketing) and enforcing actions that reduce causes of obesity (e.g., policy changes, regulations and laws) are likely to have strong impacts on reducing the obesity crisis (82).

### Anti-Obesity Medications

Pharmacotherapy is recommended for those whose BMI ≥30 (or a BMI ≥27 with comorbid conditions) and are unable to lose weight using lifestyle modification alone (83). The U.S. FDA (Food and Drug Administration) approved some new pharmacotherapy drugs for short-term obesity treatment (**Table 1**) and since Lorcaserin was withdrawn, there are only four [Naltrexone-Bupropion (Contrave), Orlistat (Xenical, Alli), Liraglutide (Saxenda) and Phentermine-Topiramate (Qsymia)] approved in addition to Gelesis which is now the fifth, have been approved for long-term use (84, 86, 87). The FDA also approved the MC4R agonist-Setmelanotide for use in individuals with severe obesity due to either POMC, PCSK1 (proprotein convertase subtilisin/kexin type 1), or LEPR (leptin receptor) deficiency at the end of 2020 (85).

**Table 1:** Prescription medications approved for obesity treatment.

Weight-loss medication	Approved for	How it works
Orlistat (Xenical) Available in lower dose without prescription (Alli)	Adults and children ages 12 and older	Works in the gut to reduce the amount of fat the body absorbs from food
Liraglutide (Saxenda) Available by injection only	Adults	May decrease hunger or increase feelings of satiation. A lower dose under a different name of Victoza was approved to treat T2DM.
Phentermine-Topiramate (Qsymia)	Adults	A mix of topiramate, which is used to treat migraine headaches or seizures, and phentermine, which lessens appetite. May decrease hunger or increase feeling of satiation.
Naltrexone-Bupropion (Contrave)	Adults	A mix of naltrexone and bupropion. May decrease hunger or increase feelings of satiation.
Gelesis (Plenity) (84)	Adults	The gel pieces increase the volume and elasticity of the stomach and small intestine contents, contributing to a feeling of fullness and inducing weight loss.
Setmelanotide (Imcivree) (85)	Adults and children ages 6 and older	An agonist of the MC4R, used in individuals with severe obesity due to either POMC, PCSK1, or LEPR deficiency, and should not be used for other types of obesity such as general obesity.
Other medications that curb your desire to eat include •Phentermine (Adipex, Superenza) •Benzphetamine (Regimex, Didrex) •Diethylpropion (Tenuate) •Phendimetrazine (Bontril PDM)	Adults	Increase chemicals in the brain to make depress feelings of hunger or increase feelings of satiation.

In addition, 11 different components have been identified from 54 families of the plants to have anti-obesity potential. These families include Celastraceae, Zingiberaceae, Theaceae, Magnoliaceae, and Solanaceae (88). Traditional Chinese medicine delivers unique solutions to treat obesity, such as regulating fat metabolism, enhancing hormone level, regulating intestinal microflora, among other pathways (89). These findings are helpful for selection of herbal medicine or traditional Chinese medicine for further research.

### Bariatric Surgery

For individuals with a BMI > 40 or BMI > 35 with comorbidities who are unable to lose weight by lifestyle modifications or pharmacotherapy bariatric surgery or weight loss surgery is another option (83). Standard bariatric operations, including BPD (Bilio-pancreatic diversion), SG (sleeve gastrectomy), RYGB (Roux-en-Y gastric bypass), and AGB (adjustable gastric banding), benefits individuals׳ metabolic profiles to varying degrees (90). Studies reported that the benefits of bariatric surgery go beyond just losing weight. Bariatric surgery reduces chronic inflammation involved in obesity and alters biomarkers, the gut microbiota, and long-term remission for T2DM (91–93). Take RYGB for example, in human subjects, overall gut microbial richness increased after RYGB surgery (94). Further analysis revealed RYGB contributed to increase of expression of some specific white adipose tissue genes, upregulation of genes central to the transforming growth factor-β signaling pathway, and remarkable downregulation of genes involved in metabolic pathways and inflammatory responses (95). Decrease of serum leptin levels, which are associated with leaned BMI, typically results from bariatric surgery. Interestingly, those women who had a higher presurgical baseline leptin level were easier to remain the post-procedure weight loss, while those with a lower presurgical baseline level were easier to regain the weight. There is a correlation between the baseline leptin level and alterations in body mass, BMI, as well as total weight loss although the success degree of surgery cannot be predicted by a patient’s serum leptin level (96).

### Fecal Microbiota Transplantation

FMT has attracted considerable research interest recently in the treatment of obesity (97). There are promising indications that FMT of microbes from healthy individuals into patients with obesity may be affected in weight loss and maintenance. In a groundbreaking key study, Ridaura et al. transplanted fecal slurries from human twins discordant for obesity into germ-free mice (98). The mice with obese individuals’ microbiota successfully developed obesity, while those with healthy individuals’ microbiota remained lean. The sequencing results of mice post-procedure stool samples showed that the human microbiomes were successfully infused, indicative of the transfer of functions related to the obese or lean microbial communities, respectively (98). Promising studies in humans are also being attempted: Vrieze et al. were able to improve microbial diversity and insulin sensitivity in obese, diabetic adult males after the transplantation with the taxa from lean donors (99). An increase was observed in butyrate-producing bacteria and Bacteroidetes, indicative of a shift toward a leaner phenotype related microbial community. While in early stages, FMT may be an option for replacing obesogenic microbial communities (100).

## Summary and Conclusions

The global prevalence of obesity has nearly tripled since 1975 and continues to grow at an exponential rate. Obesity has become the number one lifestyle-related risk factor for premature death. As such, public health policies focused on reducing and treating obesity must be developed (17). The WHO “Global Action Plan for the Prevention and Control of Noncommunicable Diseases 2013-2020” defines strategies to prevent further increase in obesity prevalence, but progress so far has been slow (101). However, with the identification of the main obesity causes the modulating factors, the challenge remains is to translate them into effective actions.

Epigenetic modifications and interactions between our genes and environment have strong influences on human health and disease. Increasing evidence is revealing the involvement of epigenetics in obesity prevalence (9). Propensity for obesity can result from the effects of environmental factors, such as nutrition and lifestyle to the epigenetic remodeling of the early postnatal development, and parental gametes. Epigenetic marks could also significantly affect the obesity risk of the child and thus be transmitted trans-generationally (11). This epigenetic ‘memory’ may help explain our lack of evidence for genetic heritability in obesity and other diseases. A foundational knowledge of the mechanisms of epigenetic inheritance is of great importance for treating and preventing obesity. Exploration of epigenetic changes is a key for predicting disease trajectories and choosing effective treatment. The reversible characteristic of these modifications makes them ideal targets for epigenetic treatment, and promising “epigenetic drugs” for therapies of obesity are already in the marketplace or in various stages of development (102). These types of therapies include DNA methyltransferase inhibitors (DNMTis), protein arginine methyltransferase inhibitors (PRMTis), histone acetyltransferase inhibitors (HATi), histone deacetylase inhibitors (HDACi), sirtuin-activating compounds (STACs) and histone demethylating inhibitors (HDMis) (103).

Microbiome research holds much promise for treating pandemics such as obesity and diabetes. On-going developments in technology and bioinformatics of microbiology are increasingly allowing for the development of a microbiome-manipulating capsule to favor a healthy, lean, and insulin-sensitive profile, but this is still an area of active research (8, 104). More targeted therapies will also become possible as we increase our understanding of microbial metabolites, allowing for clinal treatment of inflammation, weight gain and insulin resistance, and ultimately preventing the progression to obesity.

In conclusion, improved understanding of the various dimension of obesity, including propensity to regain lost weight, interindividual differences in pathogenesis, and response to therapy, is needed for developing effective as well as cost-effective interventions. The insights will in turn benefit the related health complications such as incidence of diabetes. More research is required to identify behavioral modification that are effective and available to people from diverse backgrounds. More studies were performed to develop more effective and safer medications to help obese people lose body weight and maintain a healthy weight for long term. Moreover, we must devote greater efforts and resources to the prevention of obesity in children as well as adults. Prevention is a key as treatment alone is not very effective and cannot well reverse the epidemic of obesity for long term.

## Author Contributions

HL and XL conceived and wrote the manuscript. All authors contributed to the article and approved the submitted version.

## Funding

This project was funded by grants from the Zhejiang Provincial Medical Science and Technology Program (2020KY166).

## Conflict of Interest

The authors declare that the research was conducted in the absence of any commercial or financial relationships that could be construed as a potential conflict of interest.

## Publisher’s Note

All claims expressed in this article are solely those of the authors and do not necessarily represent those of their affiliated organizations, or those of the publisher, the editors and the reviewers. Any product that may be evaluated in this article, or claim that may be made by its manufacturer, is not guaranteed or endorsed by the publisher.
